# Visual phenomenology in schizophrenia and post-traumatic stress disorder: an exploratory study

**DOI:** 10.1192/bjo.2022.544

**Published:** 2022-07-25

**Authors:** Deborah Wearne, Jeremiah Ayalde, Guy Curtis, Aarethi Gopisetty, Amit Banerjee, Peter Melvill-Smith, Kenneth Orr, Leon Rajanthiran, Flavie Waters

**Affiliations:** Department of Psychiatry, Faculty of Medicine, Dentistry and Health Sciences, University of Western Australia, Perth, Western Australia, Australia; Department of Psychology, Faculty of Medicine, Dentistry and Health Sciences, University of Western Australia, Perth, Western Australia, Australia; Early Psychosis Program Perth, headspace National Youth Mental Health Foundation, Melbourne, Victoria, Australia; Department of Psychiatry, Western Australia Department of Health, Perth, Western Australia, Australia; Department of Psychiatry, St John of God Health Care, Perth, Western Australia, Australia; School of Psychological Science, University of Western Australia, Perth, Western Australia, Australia

**Keywords:** Visual hallucinations, trauma, PTSD, schizophrenia, dissociation

## Abstract

**Background:**

Visual experiences such as hallucinations are commonly reported by people with psychosis, psychological trauma and dissociative states, although questions remain about their similarities and differences. For diagnostic and therapeutic purposes, clinical research must better delineate and compare the characteristics of these experiences in post-traumatic stress disorder (PTSD) and in schizophrenia.

**Aims:**

To compare visual phenomena and dissociation in participants with a primary psychotic illness and those with a trauma diagnosis.

**Method:**

A quantitative group design study comparing visual phenomena in three participant groups who also have a history of hearing voices: schizophrenia and no trauma history (*n* = 19), PTSD with dissociation (*n* = 17) and comorbid schizophrenia and PTSD (*n* = 20). Validated clinical measures included the North-East Visual Hallucination Interview, PTSD Symptoms Scale Interview, Clinician Administered Dissociative States Scale, Psychotic Symptoms Rating Scales and Positive and Negative Syndrome Scale.

**Results:**

There was a remarkable similarity in visual experiences, including rates of complex visual hallucinations, between the three diagnostic groups. There were no significant differences in the severity or components of distress surrounding the visual experiences. Dissociation predicted visual hallucination severity for the comorbid schizophrenia and PTSD group, but not for PTSD or schizophrenia alone.

**Conclusions:**

Visual experiences in PTSD can include visual hallucinations that are indistinguishable from those experienced in schizophrenia. Multimodal hallucinations are frequently observed in both schizophrenia and PTSD. A model for visual hallucinations in PTSD is suggested, following two separate neurobiological pathways based on distinct responses to trauma.

Visual hallucinations are well recognised in psychiatry but there is a lack of knowledge about the phenomenology of visual experiences, particularly regarding the relationship with trauma and dissociation.^1,2^ Despite a comprehensive literature showing correlations between traumatic life events and psychotic symptoms,^3^ there is a lack of research on visual experiences in trauma-exposed populations.^1^

Visual hallucinations are visual perceptions formed without an external stimulus^1^ and in schizophrenia they most commonly co-occur with auditory hallucinations.^4^ Flashbacks in post-traumatic stress disorder (PTSD) involve involuntary, distressing visual imagery, which can vary from images through to true hallucinations.^5^ These two experiences may form clinically indifferentiable constructs, as visual hallucinations can exhibit striking similarities with the intrusive visual imagery experienced in PTSD flashbacks.^6^

There are various contemporary models that strive to explain the interface between flashbacks and visual hallucinations. In the top-down model, the brain is theorised to generate predictions of sensory data and to compare this ‘best guess’ with incoming sensory data.^7^ The active construction, rather than direct reflection, of the outside world suggests a continuity between visual hallucinations and normal perception.^8^ Flashbacks may be a form of intrusive mental imagery that may manifest in ‘true’ visual hallucinations, dependent on the degree of peri-traumatic neural insult.^9^ This interpretation bears similarity to the neurocognitive failure to contextualise sensory representations of a relived traumatic event as proposed in the revised dual representation theory.^10^

This study aims to contrast and compare visual phenomenology across three diagnostic groups of individuals who also hear voices: those with schizophrenia (SCZ), PTSD with dissociation (PTSD) and both diagnoses (SCZ + PTSD). The relationships between trauma diagnosis, present-state dissociation and the experience of visual hallucinations are also examined. The goal of this research is to contribute to knowledge regarding visual experiences in schizophrenia and trauma.

## Method

### Participants

The study included 56 participants recruited from 5 in-patient and community mental health services in Perth, Western Australia. Inclusion criteria were: (a) a diagnosis of schizophrenia, PTSD with dissociation or both, established by the treating psychiatrists according to DSM-5 criteria^11^ and guided by response on clinical interviews, including the PTSD Symptoms Scale Interview (PSSI-5, see below); (b) experience of hearing voices; (c) age >18 years; and (d) capacity to give informed consent. Exclusion criteria included involuntary status and comorbid substance misuse.

The inclusion criterion of hearing voices was a purposeful strategy as visual hallucinations rarely occur on their own and tend to co-occur with another modality, usually auditory hallucinations.^4^ By using ongoing voice-hearing as a selection criterion, the study was designed to capture existing participants’ vulnerability to hallucinations and thus maximise recruitment. Studies on visual hallucinations in psychosis have used a similar recruitment strategy owing to the co-occurrence of auditory hallucinations.^12,13^

The study involved a correlational group design, where comparisons were made regarding quantitative data on visual phenomena and other symptoms, using validated measures. Patient selection was not random. This research involved discussion of potentially significant trauma, therefore from an ethical trauma-informed viewpoint, the clinical psychiatrist recruited known patients with chronic, stable illness and administered the questionnaires. Clinicians had extensive clinical experience (>20 years). All patients seen in treatment over a 6-month period who met the inclusion criteria were asked about their willingness to take part. Four patients from three sites refused to be involved owing to their personal concerns of destabilising their illness.

### Measures

#### North-East Visual Hallucination Interview (NEVHI)

The NEVHI is a validated, semi-structured 14-item scale comprised of three subscales:^14^

- phenomenology (section 1), which includes six yes/no questions about simple and complex visual hallucinations, illusions, misperceptions and feelings of a ‘presence’
- severity (section 2), based on Likert-scale ratings of the frequency and duration of visual hallucinations, which are then calculated multiplicatively with a ‘severity factor’ derived from the participant's visual phenomenology reported in NEVHI section 1 (a score of 4 denotes complex visual hallucinations; 3, illusion; 1, simple visual hallucinations, presence or passage)
- distress (section 3), which measures the emotional and cognitive distress and behavioural response that accompany participants’ experience of visual phenomena; the total score on this section is calculated only for participants who have had recurrent visual hallucinations within the past month.

The NEVHI was chosen for its use in previous studies examining visual hallucinations in psychiatric disease.^15,16^

#### PTSD Symptoms Scale Interview (PSSI-5)

The PSSI-5 is a validated, semi-structured 17-item scale to assess PTSD symptomatology and symptom severity.^17^ This scale was also used to determine whether participants with schizophrenia qualified for the SCZ + PTSD group in absence of an established PTSD diagnosis. A PSSI-5 total score > 23, indicating significant PTSD symptomatology, prompted reallocation from the SCZ to the SCZ + PTSD group for five participants.

#### Psychotic Symptoms Rating Scales (PSYRATS), hallucinations subscale

The PSYRATS is a validated clinical instrument to assess symptoms of psychosis, including auditory hallucinations.^18^ Only the hallucinations subscale was used in this study.

#### Clinician Administered Dissociative States Scale (CADSS), subjective items only

The CADSS is a validated 23-item clinical instrument used to measure present-state dissociative symptoms under close guidance of a clinician.^19^ The subjective items of the CADSS are divided into three subscales: amnesia (2 items, 14 and 15), derealisation (12 items, 1, 2, 8–13, 16–19) and depersonalisation (5 items, 3–7).

#### Positive and Negative Syndrome Scale (PANSS)

The PANSS is a widely used validated 28-item instrument for the dimensional assessment of psychosis symptoms and their severity on a 7-point scale; dimensions include positive, negative and general psychopathology.^20^

### Ethics approval

Written informed consent was obtained from all participants. The authors assert that all procedures contributing to this work comply with the ethical standards of the relevant national and institutional committees on human experimentation and with the Helsinki Declaration of 1975, as revised in 2008. All procedures involving human participants/patients were approved by St John of God Hospital (ID 1459) and Joondalup Health Campus (ID 1834) Research Ethics Committees and the National Health and Medical Research Council (NHMRC) (DW01847).

### Statistical analyses

All statistical analyses were performed using the Statistical Package for the Social Sciences (SPSS), version 28 (for Windows). Segregated diagnostic groups allowed for independence of observations. Data were visually inspected for normal distribution and deemed satisfactory to fulfil assumptions for parametric tests. Demographics, clinical variables, and mean and total psychometric scores were analysed with analysis of variance (ANOVA) and Tukey–Kramer *post hoc* tests (continuous variables) or chi-square (categorical variables). Correlations were examined using Pearson's product.

## Results

### Demographic and clinical characteristics

Study participants (*n* = 56) were categorised into three groups: those with a diagnosis of schizophrenia only (*n* = 19), those with PTSD with dissociation only (*n* = 17) and those with a dual diagnosis of schizophrenia and PTSD (*n* = 20). Table 1 shows that statistical tests (chi-squared, ANOVA) revealed no significant differences in demographic variables, including gender and age, among the three diagnostic categories.

**Table 1:** Participant demographics and clinical measures

	Diagnostic group				
	SCZ (*n* = 19)	PTSD (*n* = 17)	SCZ + PTSD (*n* = 20)	Statistical tests	*P*
Age, years: mean (s.d.)	40.9 (13.6)	40.4 (13.5)	36.4 (13.1)	*F*(2,53) = 0.65	0.52
Gender				*χ*^2^(2) = 2.12	0.34
Male, *n*	11	6	8		
Female, *n*	8	11	12		
PSSI-5 total, mean (s.d.)	^−^	61.4 (13.9)	50.4 (18.1)	*t*(35) = 2.04	**0**.04*
CADSS, mean (s.d.)					
Total score	13.8 (9.8)	34.5 (12.7)	23.1 (14.6)	*F*(2,53) = 11.77	**<0**.001*
Amnesia	1.9 (2.5)	4.1 (2.9)	2.9 (3.1)	*F*(2,53) = 2.54	0.08
Depersonalisation	3.6 (3.5)	12.6 (4.4)	6.9 (5.2)	*F*(2,53) = 18.24	***<*0**.001*
Derealisation	8.3 (5.7)	17.8 (7.2)	13.4 (8.2)	*F*(2,53) = 7.39	***<*0**.001*
PSYRATS total, mean (s.d.)	34.3 (7.5)	32.9 (6.0)	30.7 (8.8)	*F*(2,53) = 1.12	0.33
PANSS, mean (s.d.)					
Positive	21.6 (7.1)	18.8 (5.8)	24.5 (7.9)	*F*(2,53) = 3.05	**0**.05*
Negative	25.7 (6.3)	22.4 (7.5)	26.1 (6.5)	*F*(2,53) = 1.59	0.21
General	47.7 (12.8)	56.3 (13.1)	59.2 (15.2)	*F*(2,53) = 3.61	**0**.03*
NEVHI, mean (s.d.)					
Section 1 Phenomenology	3.1 (2.0)	4.0 (1.5)	4.1 (2.0)	*F*(2,53) = 1.56	0.21
Section 2 Severity	9.5 (11.6)	22.5 (18.5)	19.1 (19.5)	*F*(2,48) = 2.65	0.08
Section 3 Distress	10.2 (4.3)	9.6 (2.7)	10.5 (3.8)	*F*(2,36) = 0.21	0.80

SCZ, schizophrenia; PTSD, post-traumatic stress disorder; PSSI-5, PTSD Symptoms Scale Interview; CADSS, Clinician Administered Dissociative States Scale; PSYRATS, Psychotic Symptoms Rating Scales; NEVHI, North-East Visual Hallucination Interview.

* Denotes significance at *P ≤* 0.05.

### Trauma and dissociation measures

An independent-samples *t*-test showed significantly higher PTSD symptoms in the PTSD-only group compared with the SCZ + PTSD group, as reported by mean PSSI-5 total scores (Table 1). Trauma experiences are included in Table 2.

**Table 2:** Characteristics of trauma experienced by participants

	Diagnostic group			
	PTSD (*n* = 17)	PTSD + SCZ (*n* = 20)	*t*(35)	*P*
Childhood sexual abuse, *n*	11	10		
Childhood physical/emotional trauma, *n*	5	6		
Adult physical/emotional trauma, *n*	1	4		
Time since trauma experience, years: mean (s.d.)	30.53 (18.09)	25.70 (14.59)	0.90	0.32

PTSD, post-traumatic stress disorder; SCZ, schizophrenia.

A *post hoc* test on mean CADSS scores (Table 1) showed that participants with PTSD alone experienced significantly more dissociative symptoms than participants with either SCZ + PTSD (*P* = 0.022) or schizophrenia alone (*P* < 0.001).

Lastly, *post hoc* tests on PANSS domains (Table 1) showed that participants with SCZ + PTSD had higher ratings of general psychopathology than those with schizophrenia alone (*P* = 0.03), supporting the understanding that comorbid diagnoses are likely to be associated with higher rates of symptomatology.^21^ However, the difference in positive psychotic symptoms was observed only for the SCZ + PTSD group, with higher positive symptoms than for those with PTSD only (*P =* 0.04). No significant difference in mean PANSS-Positive scores was observed between the SCZ + PTSD and SCZ groups (*P* = 0.41).

### Hallucination measures

Severity and frequency of auditory hallucinations were similar between the three groups as measured on the PSYRATS (Table 1). Within this study population reporting auditory hallucinations, 91% reported visual hallucinations.

Analyses of visual hallucination phenomenology on the NEVHI section 1 showed no significant differences between the three groups (Table 3). As a population, participants were more likely to identify the experience of complex visual hallucinations (79%) compared with illusions (45%) (*χ*^2^(1) = 8.15, *P* = 0.004) and simple hallucinations (45%) (*χ*^2^(1) = 12.32, *P* < 0.001), as reported on the NEVHI section 1.

**Table 3:** Visual hallucination phenomenology: North-East Visual Hallucination Interview (NEVHI) section 1 and clinician-rated

	Diagnostic group					
	SCZ (*n* = 19), mean (s.d.)	PTSD (*n* = 17), mean (s.d.)	SCZ + PTSD (*n* = 20), mean (s.d.)	*F*(2,53)	*P*	Proportion experiencing the symptoms (%)
NEVHI section 1						
Complex visual hallucinations	0.6 (0.5)	0.9 (0.2)	0.8 (0.4)	2.68	0.08	79
Illusions	0.5 (0.5)	0.4 (0.5)	0.5 (0.5)	0.43	0.65	45
Presence	0.7 (0.5)	0.8 (0.4)	0.9 (0.4)	0.73	0.49	77
Passing shadow	0.6 (0.5)	0.8 (0.4)	0.7 (0.5)	1.26	0.29	70
Other visual experiences	0.4 (0.5)	0.7 (0.5)	0.6 (0.5)	1.54	0.22	57
Simple visual hallucinations	0.3 (0.5)	0.4 (0.5)	0.6 (0.5)	1.66	0.20	45
Total	3.1 (2.0)	4.0 (1.5)	4.0 (2.0)	1.57	0.22	91
Clinician-rated						
Complex visual hallucinations	0.4 (0.5)	0.7 (0.5)	0.6 (0.5)	1.92	0.16	48
Simple visual hallucinations	0.2 (0.4)	0.5 (0.5)	0.4 (0.5)	1.60	0.21	36
Illusions	0.8 (0.4)	0.5 (0.5)	0.7 (0.5)	0.93	0.40	59

SCZ, schizophrenia; PTSD, post-traumatic stress disorder.

As supplementary information, participants were asked to briefly describe their visual experiences. Understandably, the trauma-exposed groups (PTSD and SCZ + PTSD) reported higher trauma-related visual hallucinations (*χ*^2^(2) = 6.91, *P* = 0.03). In addition, clinicians were asked to subjectively classify participants’ experiences into any combination of complex hallucinations, simple hallucinations, illusions or ‘none of the above’. There were no significant differences in clinician-rated phenomenology between the three groups (Table 3).

### Severity of visual experiences

**Fig. 1:**
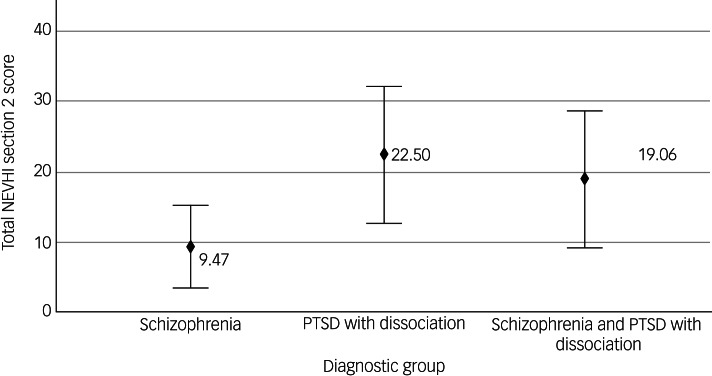
Visual hallucination severity scores on section 2 of the North-East Visual Hallucination Interview (NEVHI): means by diagnostic group. Error bars show 95% CIs. PTSD, post-traumatic stress disorder.

Analyses of the NEVHI section 2 data showed no significant differences in mean visual hallucination severity scores between diagnostic groups (*F*(2,48) = 2.65, *P* = 0.08). The means plot for NEVHI section 2 is shown in Fig. 1.

### Emotional, behavioural and cognitive symptoms of distress

The NEVHI section 3 measured items of emotional, behavioural and cognitive distress for ongoing, recurrent visual experiences. Mean total distress scores showed no significant differences between groups (*F*(2,36) = 0.21, *P* = 0.80). Visual phenomena were equally likely to be experienced as real, irritating, distressing, difficult to ignore and associated with ‘acting out’ and fears of ‘losing one's mind’ across all three diagnostic groups (Table 4).

**Table 4:** North-East Visual Hallucination Interview (NEVHI) section 3, per-item scores

	Diagnostic group				
	SCZ (*n =* 17), mean (s.d.)	PTSD (*n =* 16), mean (s.d.)	SCZ + PTSD (*n* = 18), mean (s.d.)	*F*(2,48)	*P*
NEVHI section 3					
Irritating	1.9 (1.1)	1.6 (1.0)	1.4 (1.2)	0.69	0.51
Distressing	2.0 (1.1)	1.9 (0.9)	1.8 (1.0)	0.14	0.87
Losing my mind	1.7 (1.2)	1.6 (1.0)	1.7 (0.9)	0.05	0.95
Believe it is real	1.7 (1.0)	1.4 (0.9)	1.5 (0.9)	0.36	0.70
Acting out	0.4 (0.7)	0.9 (1.0)	0.9 (0.9)	1.69	0.20
Unable to ignore	2.4 (1.0)	2.3 (0.8)	1.9 (0.8)	1.37	0.27

SCZ, schizophrenia; PTSD, post-traumatic stress disorder.

### Correlations

Age and all psychometric scores were correlated separately for each of the diagnostic groups (supplementary Appendix 1, available at https://doi.org/10.1192/bjo.2022.544).

The auditory hallucinations severity measure, PSYRATS, showed clear evidence of significant correlation with CADSS scores in all three diagnostic groups (Table 5). For visual hallucinations explored through the NEVHI, correlations with CADSS dissociation were observed only in the SCZ + PTSD group, for whom severity scores (section 2) and emotional, cognitive or behavioural associations (section 3) correlated significantly. Of note, participants in the PTSD group showed no such correlation. A correlation was also observed between PTSD symptom severity and visual hallucinations severity in the SCZ + PTSD group (*R*(16) = 0.46, *P* = 0.05). For all three groups, PANSS scores did not correlate with any of the three NEVHI domains.

**Table 5:** NEVHI–CADSS and PSYRATS–CADSS correlations,^a^ by diagnostic group

	SCZ	PTSD	SCZ + PTSD
NEVHI section 1–CADSS total	0.31 (*P* = 0.21)	−0.04 (*P* = 0.88)	0.35 (*P* = 0.13)
NEVHI section 2–CADSS total	0.42 (*P* = 0.10)	0.17 (*P* = 0.52)	0.57* (*P* = 0.01)
NEVHI section 3–CADSS total	0.47 (*P* = 0.15)	0.15 (*P* = 0.61)	0.62* (*P* = 0.02)
PSYRATS–CADSS total	0.47* (*P* = 0.05)	0.57* (*P* = 0.02)	0.65* (*P* = 0.002)

NEVHI, North-East Visual Hallucination Interview; CADSS, Clinician Administered Dissociative States Scale; PSYRATS, Psychotic Symptoms Rating Scales; SCZ, schizophrenia; PTSD, post-traumatic stress disorder.

a. All correlations as Pearson's *r*.

* Denotes significance at *P* ≤ 0.05.

## Discussion

This research is one of the first efforts at assessing and comparing visual phenomena in schizophrenia and PTSD. Visual experiences, including visual hallucinations, occurred at remarkably similar rates, with similar severity and in a similar manner in all participants who heard voices, despite their diagnosis.

Given that the inclusion criterion of hearing voices increases incidence of other modalities of hallucination,^4^ the high rates of visual experiences (91%) comparative to previous research^1^ can be understood. This criterion also reduces the generalisability of the results from broad diagnoses of schizophrenia and PTSD to the subgroups who also hear voices.

The components of distress (emotional, cognitive and behavioural) associated with the visual phenomena were experienced similarly between diagnostic groups. Visual experiences were equally likely to be seen as real, intrusive, distressing, irritating, fear-inducing and resulting in acting out behaviour. The similarity of distress measures in the SCZ, PTSD and SCZ + PTSD groups suggests that dissociative and psychotic visual experiences engender similar appraisals and behavioural consequences regardless of aetiology. In this research, participants in the trauma-exposed groups described significantly higher rates of trauma-related visual hallucinations, suggesting content consistent with the experience of flashbacks.

### New concepts: multimodal hallucinations

Emerging evidence in both schizophrenia and PTSD suggests that visual hallucinations rarely occur alone and are instead present in individuals also experiencing auditory hallucinations.^22^ The term ‘multimodal hallucinations’ refers to hallucinations in multiple modalities, occurring either serially or simultaneously.^23,24^ Although auditory hallucinations remain the most prevalent type of hallucination in schizophrenia there is an approximately 90% probability of co-occurring hallucinations in other modalities.^15,23^

Our research adds to the evidence that auditory hallucinations are likely to be associated with visual experiences, including visual hallucinations. These findings include PTSD with dissociation in experiencing hallucinations in multiple modalities. This evidence has clinical relevance, emphasising the importance of considering multiple modalities when assessing patients who are hearing voices. A limitation of our study design was that the methodology did not explore temporal or subject relationships between visual hallucinations and auditory hallucinations. For future research, a gold-standard clinical instrument measuring these aspects of multimodal hallucinations would be welcomed.

### The role of dissociation in visual experiences

The role of dissociation in the experience of hallucinations in both PTSD and psychosis has been previously explored in the auditory but not visual modality, with strong findings that suggest auditory hallucinations are uniquely mediated by dissociation in trauma-exposed populations.^25,26^

In the present research the evidence regarding the role of dissociation varied significantly when comparing auditory and visual hallucinations. The only diagnostic group that showed a correlation between visual hallucinations and dissociation was SCZ + PTSD. As PANSS scores did not correlate with visual hallucination measures in the SCZ + PTSD group, it is less likely that this could be attributed to illness severity. In contrast, there were significant correlative findings across all three diagnostic groups for auditory hallucinations and dissociation.

There are several potential explanations for these differences. First, there was the limitation of smaller sample size, which meant that sampling variability and multiple statistical comparisons potentially affected the statistical power of analyses, and a larger-scale study might have revealed different results.

However, following the principles of multimodal hallucinations, visual hallucinations were initially expected to bear a similar relationship to dissociative symptoms as their auditory counterparts. Our research showed two differing patterns of relationship: in the SCZ + PTSD group, there was a correlation between dissociation and visual hallucinations with lower rates of PTSD symptoms, whereas for the PTSD-only group, there was no correlation with dissociation despite significantly higher rates of PTSD symptoms. This finding lends to the possibility of two different types of visual hallucination in PTSD: visual hallucinations in flashbacks versus chronic dissociative hallucinations.

### Visual experiences in PTSD: a proposal for two aetiologies

Clinically, flashbacks are considered a core diagnostic feature of PTSD and are present from the beginning of the illness process.^27^ In contrast, chronic dissociation tends to develop along a course of repeated traumatic exposures, as seen commonly in childhood trauma and complex PTSD.^28^

Flashbacks in PTSD are physiologically associated with the ‘fight or flight’ response, which is based on the sympathetic nervous system and linked to pupillary dilation and visual focus.^29^ Pathologically, at a time of severe psychological insult, incoming sensory details such as visual information may be improperly encoded into memory, subsequently generating flashbacks.^30^

In contrast, chronic dissociative hallucinations are more likely linked to the ‘freeze’ or ‘play dead’ response, which is based on the parasympathetic nervous system.^31^ It is theorised that dissociation may form an alternative learned pathway, such that parasympathetic networks override physiologically during the re-experiencing phenomenon.^31^ The accompaniment of dissociation with visual hallucinations in trauma is likely neurophysiological, cognitively reinforced over time and emergent in stress, as in dissociative auditory hallucinations.^25^ In this way, visual hallucinations in the dissociative state may occur secondary to downstream signals of hallucinations in the auditory modality.^32^

The theoretical differences between flashbacks and dissociative visual hallucinations are reflected in our findings. Despite both groups experiencing high levels of dissociation, the PTSD group had higher overall PSSI-5 scores, suggesting the possibility of a component of sympathetic overdrive in stress, and no link between visual hallucinations and dissociation. In contrast, the SCZ + PTSD group showed a clear relationship between visual hallucinations and dissociation, but significantly lower acute PTSD symptomatology.

Although this is an exploratory study, one possible explanation of our observations is that there are two separate aetiologies of PTSD visual re-experiencing: (a) a direct, contextualised, flashback visual memory, on a stress-mediated spectrum of visual hallucinations; and (b) a learned, multisensory neurocognitive process in times of stress, predominantly auditory over visual, and involving dissociative phenomena such as contextual fragmentation (summarised in Table 6). Our research findings present hypotheses that require closer evaluation in further research.

**Table 6:** Visual hallucinations in post-traumatic stress disorder (PTSD) and responses to trauma

	Visual hallucinations in flashbacks	Chronic dissociative visual hallucinations
Diagnosis	PTSD	Complex PTSD
Hallucination characteristics	Intrusive trauma-specific flashbacks, predominately visual	Occurring with chronic dissociation, primarily auditory hallucinations but secondary visual hallucinations occur
Autonomic nervous system behaviour	Sympathetic drive: pupillary dilation and visual focus	Parasympathetic drive: ‘playing dead’ survival focus
Behavioural imperative	‘Fight or flight’	‘Freeze’
Trauma history	Specific traumatic event	Chronic and repetitive exposures – childhood traumas common
Current research	Higher PTSD symptoms; no correlation with dissociation	Lower PTSD symptoms; positive correlation with dissociation

### Limitations

There were some significant limitations in this study.

The inclusion criterion of hearing voices limited the generalisability of findings from schizophrenia and PTSD diagnoses to the subgroups who also hear voices.

The NEVHI was originally designed to assess visual experiences in neuropsychiatric populations, such as those with Lewy body dementia or Parkinson's disease.^14^ Although they are validated instruments, they may not be sensitive to the aspects of visual hallucinations that differ between the groups. There remains a significant lack of validated screening instruments for visual experiences in general psychiatry.^33^

The small sample size remained an issue, affecting the data's generalisability to the broader population. There is some risk of type 1 errors with multiple statistical comparisons, but because of the exploratory nature of the study, alpha was not adjusted.

Diagnosis depended on accurate clinical assessment, and analysing comorbidity can be challenging in already complex cases, despite the clinicians having extensive clinical experience (>20 years). Hallucinations and dissociation are complex experiences, vulnerable to contamination and difficult to operationalise.

### Clinical and research implications

Further research in the area of visual experiences would be significantly helpful to bring increased clarity to this complex problem of the experience of flashbacks and the visual phenomenology that accompanies this occurrence. There is a need for the development of more appropriate instruments to assess visual experiences in general psychiatry.

## Data Availability

The data that support the findings of this study are available from the corresponding author D.W. on reasonable request.

## Supplementary material

For supplementary material accompanying this paper visit http://doi.org/10.1192/bjo.2022.544. click here to view supplementary material
