# Supplementary material for: Visual phenomenology in schizophrenia and post-traumatic stress disorder: an exploratory study
Source: BJPsych Open. 2022 Jul 25;8(4):e143. doi: 10.1192/bjo.2022.544 (PMC9345685; doi:10.1192/bjo.2022.544)
Supplement: Supplementary file 1 [file S2056472422005440sup001.docx]

***Appendix 1 – Correlation matrices (age & clinical psychometric scores)***

**Schizophrenia**

| *R* | Age | PSSI-5 total^a^ | CADSS total | PSYRATS total | PANSS Positive | PANSS Negative | PANSS General | NEVHI S1 | NEVHI S2 | NEVHI S3 |  |
| --- | --- | --- | --- | --- | --- | --- | --- | --- | --- | --- | --- |
| Age | 1.00 | - | .28 | .43 | .45 | .17 | .23 | .42 | -.10 | .29 |  |
| PSSI-5 total^a^ | - | - | - | - | - | - | - | - | - | - |  |
| CADSS total | .28 | - | 1.00 | .47* | -.06 | -.17 | .07 | .31 | .42 | .47 |  |
| PSYRATS total | .43 | - | .47* | 1.00 | .59** | .46* | .61** | 0.10 | .003 | .16 |  |
| PANSS Positive | .45 | - | -.06 | .59** | 1.00 | .74*** | .84*** | .35 | -.12 | -.11 |  |
| PANSS Negative | .17 | - | .17 | .46* | .74*** | 1.00 | .73*** | .03 | -.19 | -.28 |  |
| PANSS General | .23 | - | .07 | .61** | .84*** | .73*** | 1.00 | .34 | .06 | -.12 |  |
| NEVHI S1 | .42 | - | .31 | 0.10 | .35 | .03 | .34 | 1.00 | .33 | .56 |  |
| NEVHI S2 | -.10 | - | .42 | .003 | -.12 | -.19 | .06 | .33 | 1.00 | .48 |  |
| NEVHI S3 | .29 | - | .47 | .16 | -.11 | -.28 | -.12 | .56 | .48 | 1.00 |  |

* *p* < .05; ** *p* < .01; *** *p* <.001 ^a^ PSSI-5 scores <23 for participants with schizophrenia (not re-allocated to SCZ+PTSD) were excluded from analysis.

| *R* | Age | PSSI-5 total | CADSS total | PSYRATS total | PANSS Positive | PANSS Negative | PANSS General | NEVHI S1 | NEVHI S2 | NEVHI S3 |  |
| --- | --- | --- | --- | --- | --- | --- | --- | --- | --- | --- | --- |
| Age | 1.00 | .44 | .35 | .58* | .07 | .09 | .17 | -.53* | -.19 | -.50 |  |
| PSSI-5 total | .44 | 1.00 | .54* | .76*** | .07 | .20 | .36 | -.13 | -.02 | -.42 |  |
| CADSS total | .35 | .54* | 1.00 | .57* | .18 | .18 | .18 | -.04 | .17 | .15 |  |
| PSYRATS total | .58* | .76*** | .57* | 1.00 | .23 | .29 | .45 | -.09 | .17 | -.48 |  |
| PANSS Positive | .07 | .07 | .18 | .23 | 1.00 | .45 | .66* | .08 | -.30 | .03 |  |
| PANSS Negative | .09 | .20 | .18 | .29 | .45 | 1.00 | .78*** | -.34 | -.16 | -.01 |  |
| PANSS General | .17 | .36 | .18 | .45 | .66** | .78*** | 1.00 | -.30 | -.30 | -.10 |  |
| NEVHI S1 | -.53* | -.13 | -.04 | -.09 | .08 | -.34 | -.30 | 1.00 | .31 | .40 |  |
| NEVHI S2 | -.19 | -.02 | .17 | .17 | -.30 | -.16 | -.30 | .31 | 1.00 | .24 |  |
| NEVHI S3 | -.50 | -.42 | .15 | -.48 | .03 | -.01 | -.10 | .40 | .24 | 1.00 |  |

**PTSD**

* *p* < .05; ** *p* < .01; *** *p* <.001

| *R* | Age | PSSI-5 total | CADSS total | PSYRATS total | PANSS Positive | PANSS Negative | PANSS General | NEVHI S1 | NEVHI S2 | NEVHI S3 |  |
| --- | --- | --- | --- | --- | --- | --- | --- | --- | --- | --- | --- |
| Age | 1.00 | .31 | .42 | .42 | .19 | .29 | .32 | -.16 | .11 | .15 |  |
| PSSI-5 total | .31 | 1.00 | .68** | .71*** | -.13 | .40 | .07 | .06 | .46* | .39 |  |
| CADSS total | .42 | .68** | 1.00 | .65** | -.07 | .35 | .15 | .35 | .57* | .62* |  |
| PSYRATS total | .42 | .71*** | .65** | 1.00 | .06 | .57* | .02 | .14 | .17 | .07 |  |
| PANSS Positive | .19 | -.13 | -.07 | .06 | 1.00 | .34 | .74*** | -.19 | -.13 | -.05 |  |
| PANSS Negative | .29 | .40 | .35 | .57* | .34 | 1.00 | .72** | -.06 | .25 | .21 |  |
| PANSS General | .32 | .07 | .15 | .02 | .74*** | .72** | 1.00 | -.16 | .20 | .27 |  |
| NEVHI S1 | -.16 | .06 | .35 | .14 | -.19 | -.06 | -.16 | 1.00 | .53* | .31* |  |
| NEVHI S2 | .11 | .46* | .57* | .17 | -.13 | .25 | .20 | .53* | 1.00 | .63* |  |
| NEVHI S3 | .15 | .39 | .62* | .07 | -.05 | .21 | .27 | .31* | .63* | 1.00 |  |

**Schizophrenia and PTSD**

* *p* < .05; ** *p* < .01; *** *p* <.001
